# A Delayed Presentation of Superior Mesenteric Artery Syndrome Treated With Robotic-Assisted Strong Procedure

**DOI:** 10.7759/cureus.67527

**Published:** 2024-08-22

**Authors:** Ashton White, Kailey Shannon, Joshua Stewart, Mujahed Laswi, Basem Soliman

**Affiliations:** 1 Department of Surgery, Texas Tech University Health Sciences Center, Amarillo, USA

**Keywords:** strong procedure, robotic-assisted surgery, wilkie's syndrome, superior mesenteric artery syndrome, superior mesenteric artery (sma)

## Abstract

Superior mesenteric artery (SMA) syndrome, also known as Wilkie’s syndrome, is a rare condition resulting from compression of the third portion of the duodenum between the aorta and the superior mesenteric artery. When symptomatic, this compression may result in nausea, vomiting, epigastric discomfort, and weight loss, requiring clinical attention and imaging to make the diagnosis. Typically, SMA syndrome presents in young females and is associated with an underlying condition such as anorexia nervosa, cachexia, postoperative development after scoliosis surgery, etc. In this report, we present the case of an atypical delayed presentation of SMA syndrome in an 84-year-old male who had epigastric pain, nausea, intermittent vomiting, and a 30-pound weight loss over two years. SMA syndrome was diagnosed on a computed tomography (CT) scan by a decreased angle between the superior mesenteric artery and abdominal aorta, and treatment with a robotic-assisted strong procedure was performed. The patient was followed postoperatively in the clinic and tolerated the procedure well.

## Introduction

Superior mesenteric artery (SMA) syndrome is a condition resulting from compression of the third portion of the duodenum between the aorta and the superior mesenteric artery. This syndrome is considered rare, with a reported prevalence between 0.013% and 0.3% [1], and is usually accompanied by nausea, vomiting, epigastric pain, and extensive weight loss. Although considered rare, SMA syndrome is believed by some authors to occur more frequently; however, those with mild compression do not present with symptoms, and the diagnosis is often delayed [2]. The physician typically needs a high index of suspicion to make a diagnosis, which can be established by clinical symptoms supported by imaging through one or more modalities (upper gastrointestinal series, computed tomography [CT] and angiography, magnetic resonance imaging [MRI], etc.). The initial approach to treatment is typically conservative management with nutrition optimization, followed by surgery if obstructive symptoms persist [3]. Below, we describe the case of an 84-year-old male presenting with epigastric pain, nausea, intermittent vomiting, and a 30-pound weight loss, which was found to have a decreased angle between the aorta and superior mesenteric artery causing compression of the third portion of the duodenum, diagnosed as superior mesenteric artery syndrome and treated with a robotic-assisted Strong procedure.

## Case presentation

**Figure 1:**
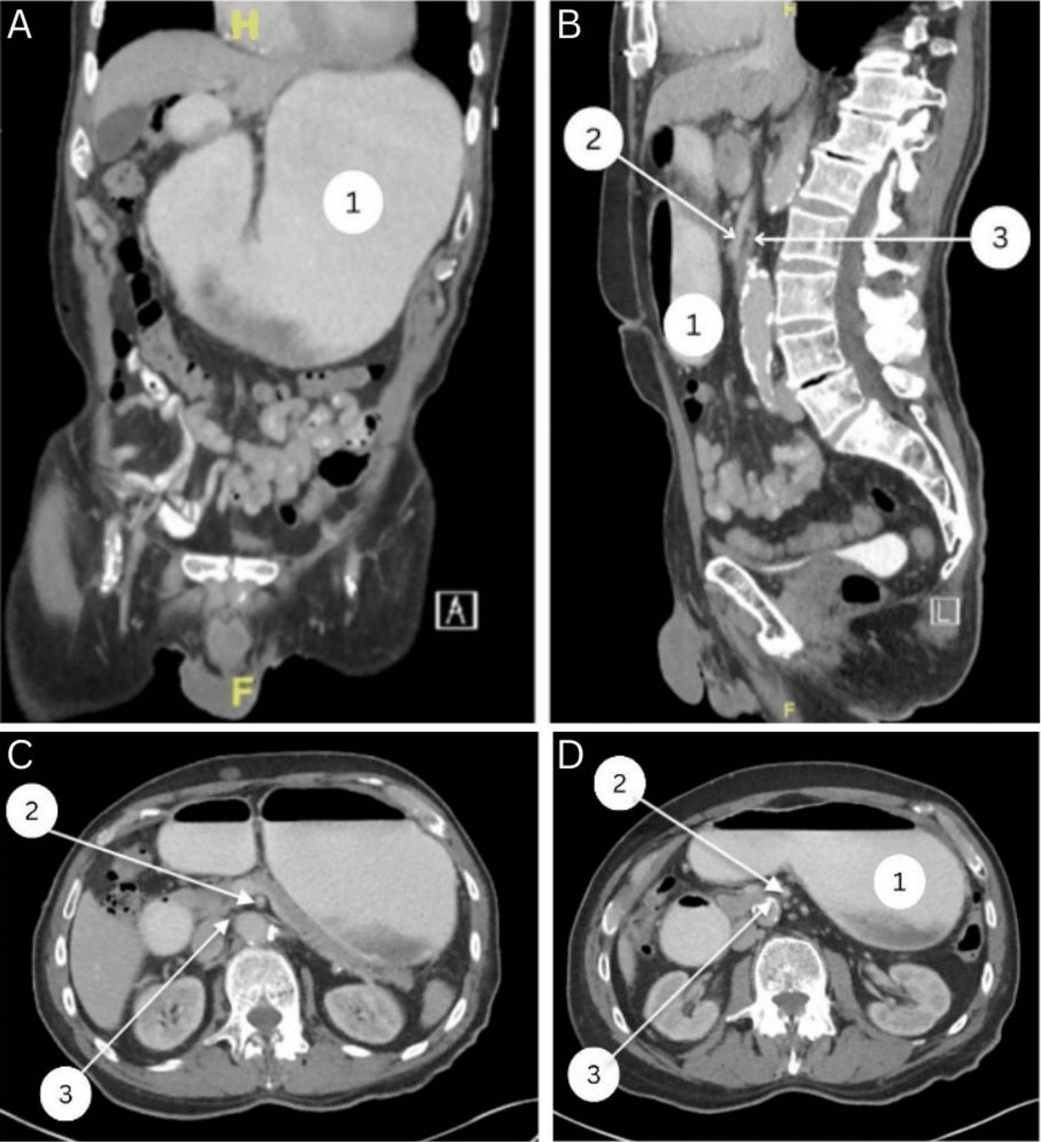
Preoperative CT images in the coronal, sagittal, and axial views (1) Distended stomach; (2) superior mesenteric artery; (3) compressed third portion of the duodenum

An 84-year-old male with a past medical history of hypertension, hyperlipidemia, and chronic gastroesophageal reflux disease presented to the emergency room with worsening abdominal pain and constipation over the last four to five days. He stated that he continued to have constipation despite the frequent use of multiple laxatives and an enema. Over the past few months, he described multiple episodes of nausea, intermittent bilious emesis, and a decreased appetite associated with a 30-pound weight loss over the last two years. He denied hematochezia, hematuria, or any other symptoms. The patient was afebrile and hemodynamically stable, with an oxygen saturation of 95% on room air. On admission, we completed laboratory testing (Table 1). A physical exam revealed a soft, non-tender abdomen with mild distention and normal bowel sounds. A computed tomography (CT) scan of the abdomen and pelvis with intravenous and oral contrast revealed marked distention of the stomach and duodenum to the level of the mid-third portion with a narrowed duodenum between the superior mesenteric artery and aorta, concerning for SMA syndrome (Figure 1). A nasogastric tube was placed on low-intermittent suction. Alternative management plans were discussed in detail, including conservative management with nutrition optimization versus surgery. Given the older age of the patient and his comorbidities, we opted to proceed with surgery, and the patient consented to a robotic-assisted Strong procedure with mobilization of the duodenum to relieve the obstruction.

**Table 1:** Laboratory values on admission

Laboratory values	Patient values	Reference ranges
Hemoglobin (g/dL)	15.6	13.5–17.5
Hematocrit (%)	45	41–50
WBC (×10^3^/mcL)	10.3	4.5–11
Platelets (×10^3^/mcL)	216	130–400
Sodium (mmol/L)	139	135–145
Potassium (mmol/L)	3.9	3.4–5
Albumin (g/dL)	4.4	3.1–4.3

We brought the patient to the operating room after obtaining informed consent for the procedure. Using a 5 mm Optiview camera in the left upper quadrant, ports were placed in the periumbilical region, right upper quadrant, and left lower quadrants of the abdomen. After establishing the pneumoperitoneum, the robot was docked, and an exploration of the peritoneal cavity revealed extensive dilation of the duodenum with a transition point in the area of the mesenteric artery/third portion of the duodenum. Complete kocherization of the duodenum using an electrocautery hook was performed with the release of the duodenum of the inferior vena cava and the retroperitoneal attachments. Then, our attention was directed to the third portion of the duodenum, which was released from the uncinate process of the pancreas. Next, the transverse colon was left cephalic, and the Treitz ligament was released using electrocautery Bovie®. The duodenum with the proximal jejunum was freed from all retroperitoneal attachments and mobilized to the right of the superior mesenteric artery. Bleeding was noted from a small arterial branch of the superior mesenteric artery, and two clips were applied. An area of serosal tear was noted in the duodenum from overdistention, which was repaired with interrupted 3-0 silk stitches. Using a bipolar device, an appropriately sized tongue of vascularized omentum was made and placed behind the superior mesenteric artery to prevent the recurrence of the SMA syndrome. Two stitches of 2-0 silk were used to fix the omentum to the retroperitoneum around the superior mesenteric artery, and 3-0 silk stitches were used to secure the proximal jejunum to the omentum. The peritoneal cavity was irrigated with warm saline, and hemostasis was achieved and confirmed. The patient tolerated the procedure without any immediate complications and was transferred to the post-anesthesia care unit (PACU) in stable condition.

The patient remained at the hospital for two weeks postoperatively under close supervision for postoperative aspiration pneumonia and respiratory failure. After discharge, he was advised to follow up in the clinic at two weeks and three months postoperatively. At his two-week postoperative appointment, our patient stated he was recovering well, tolerating his diet with no associated nausea, vomiting, fever, chills, diarrhea, or abdominal pain, and gaining weight appropriately with an expected smooth recovery and favorable prognosis.

## Discussion

SMA syndrome is a potentially life-threatening condition caused by changes in the anatomical relationship between the superior mesenteric artery anteriorly and the duodenum and aorta posteriorly [4]. While relatively rare, some of the most common etiologies identified for SMA syndrome include post-scoliosis surgery, in which lengthening of the spine increases tension and narrowing of the aortomesenteric angle [5], trauma to the spine, abdominal surgery (total proctocolectomy), neoplastic conditions or states resulting in cachexia/wasting, and anorexia nervosa resulting in loss of intra-abdominal fat, decreasing the aortomesenteric angle [6]. In the case of our patient, he had a calculated BMI of 20.96, which falls within the healthy range. He also denied a history of scoliosis with surgical correction, recent spinal trauma, or anorexia nervosa, and had no history of cancer or other neoplastic conditions.

SMA syndrome has been reported to present both insidiously and acutely. This makes diagnosing this syndrome challenging and can lead to delayed treatment and worsening of symptoms. While SMA syndrome can occur in any demographic, it typically occurs in younger adults aged 10 to 40 years old, with a higher prevalence in females, most likely due to an association with a patient’s underlying comorbid condition (i.e., weight loss from chemotherapy, anorexia nervosa, etc.) [7-8]. In a 2013-2014 study evaluating ten cases of SMA syndrome, the median age of presentation was 40 years old, with symptoms developing between 6 and 24 months [6]. Our patient’s presentation of SMA syndrome at age 84 is unique as he is well above the typical age threshold. In contrast to SMA syndrome, the pathology of the superior mesenteric artery in an elderly patient is typically related to atherosclerotic disease. These patients are in their fifth or sixth decade of life with a past medical history of peripheral artery disease, coronary artery disease, or cerebrovascular disease who present with symptoms of ischemia such as postprandial abdominal pain [9].

Treatment options for SMA syndrome are either conservative or surgical. Fluid resuscitation, correction of electrolyte imbalances, bowel rest, and nutritional optimization are typically the first lines of action. In children and young adults with a short history of symptoms, this may be a reasonable option, but in the chronic adult patient, conservative treatments often require prolonged in-hospital management and surgical correction at a later date [10]. Given our patient’s age at presentation, surgical correction was indicated to decrease the length of hospital stay and promote a faster recovery. The strong procedure is a minimally invasive procedure in which the ligament of Treitz is divided, and the duodenum is mobilized and positioned to the right of the SMA to avoid duodenal compression [11]. This procedure offers many advantages, including being less invasive and quicker, with no risk of anastomotic leak. Using the robotic platform capitalizes on efficiency, wrist articulation, and cosmetic outcomes [1].

## Conclusions

While more common in young females, SMA syndrome is a differential that should be considered in both male and female patients at the extremes of age presenting with obstructive symptoms. Varying degrees of severity and onset make this syndrome difficult to diagnose, but a high degree of clinical suspicion should warrant imaging and subsequent treatment. Treatment is typically conservative, but given an older age at presentation, surgical correction may be indicated first-line to decrease the length of hospital stay and promote a faster recovery.
